# Disseminated organ and tissue infection secondary to carbapenem-resistant *Klebsiella pneumoniae* bloodstream infection for acute lymphoblastic leukemia treated with ceftazidime–avibactam: Two case reports

**DOI:** 10.1097/MD.0000000000041195

**Published:** 2025-01-10

**Authors:** Mingxia He, Yuxia Jiang, Haiying Wu, Xiaofeng Xu, Huifang Jiang

**Affiliations:** a Department of Hematology, Tongde Hospital of Zhejiang Province, Hangzhou, P.R. China; b Department of Hematology, Hangzhou Red Cross Hospital, Hangzhou, Zhejiang, P.R. China.

**Keywords:** acute lymphoblastic leukemia, bloodstream infection, carbapenem-resistant *Klebsiella pneumoniae*, ceftazidime–avibactam, organ and tissue infection

## Abstract

**Rationale:**

Carbapenem-resistant *Klebsiella pneumoniae* (CRKP) bloodstream infections are a severe complication resulting from granulocyte deficiency following chemotherapy for hematologic malignancies and have a high mortality rate. However, reports of disseminated organ infections secondary to bloodstream infections are rare.

**Patient concerns and diagnoses:**

We report 2 cases of patients with acute lymphoblastic leukemia who both developed CRKP bloodstream infections during the granulocyte deficiency stage following chemotherapy, with 1 case of secondary bacterial liver abscess and 1 case of secondary septic arthritis.

**Interventions and outcomes:**

Based on the results of drug sensitivity testing, both patients were treated with ceftazidime–avibactam, and the infections were rapidly and effectively controlled without significant adverse effects.

**Lessons:**

Ceftazidime–avibactam exhibited satisfactory efficacy and safety in the 2 cases of disseminated organ infection secondary to CRKP bloodstream infection following chemotherapy for acute lymphoblastic leukemia.

## 1. Introduction

Patients with hematologic malignancies (HM) are highly susceptible to infections during the granulocyte deficiency phase following chemotherapy. *Klebsiella pneumoniae* is a common pathogen, and bloodstream infections due to carbapenem-resistant *K pneumoniae* (CRKP) infection have a high morbidity and mortality rate, ranging from 24% to 70%.^[1]^ Disseminated organ infections secondary to CRKP bloodstream infections are uncommon and have limited effective therapeutic agents.^[2]^ The novel third-generation cephalosporinase inhibitor ceftazidime–avibactam (CAZ–AVI), a combination of ceftazidime (CAZ) and the β-lactamase inhibitor avibactam (AVI),^[3]^ exhibits anti-CRKP activity. We report 2 cases of patients with acute lymphoblastic leukemia (ALL) who developed CRKP bloodstream infections along with secondary infections of the liver and soft tissues of the joints during the granulocyte deficiency phase. CAZ–AVI was used to effectively control the infections with no significant adverse events.

## 2. Case presentation

### 2.1. Case 1

A male patient, aged 40 years, tested positive for intestinal carbapenem-resistant Enterobacteriaceae screening. He was diagnosed with T-cell ALL in March 2015 and underwent allogeneic hematopoietic stem cell transplantation (allo-HSCT) in September 2015. He developed recurrence in October 2019 but achieved complete remission after chemotherapy. On January 8, 2021, he underwent maintenance chemotherapy with venetoclax (100 mg on days 1–7) + AA regimen (aclacinomycin 20 mg on days 1–4 + cytarabine 35 mg q12 hours on days 1–10). On + 17 days, bone marrow suppression resulted in severe granulocyte deficiency, and the neutrophil count was 0. The patient developed fever with a maximum body temperature (*T*_max_) of 40.7 °C, accompanied by pronounced chills, shivering, and obvious symptoms of sepsis. He was empirically treated with meropenem (1 g IV q8 hours, 3 days), linezolid (0.6 g IV q12 hours, 2 days), tigecycline (100 mg IV q12 hours, 2 days), and voriconazole (loading dose 6 mg/kg IV q12 hours for 1 day, maintenance dose 4 mg/kg IV q12 hours, 3 days); however, his body temperature was not controlled. Inflammatory indicators increased; CRP reached a maximum value of 266.4 mg/L and PCT reached 11.6 ng/mL. Blood culture showed CRKP (drug sensitivity test results shown in Table 1). The patient underwent antibiotic treatment with CAZ–AVI 2.5 g IV q8 hours, after which his body temperature was rapidly controlled, and inflammatory indicators returned to normal. CAZ–AVI was discontinued after 7 days, following recovery from agranulocytosis. The time to blood culture conversion was 7 days.

**Table 1:** Pathological, microbiological, and drug sensitivity testing of cases 1 and 2.

	MIC (mg/L)			
	Case 1 bloodstream	Case 1 liver tissue	Case 2 bloodstream	Case 2 synovial cavity
Ticarcillin/clavulanic acid	≥128	≥128	–	≥128
Piperacillin/tazobactam	≥128	≥128	≥128	≥128
Ceftazidime	≥64	32	–	≥64
Cefoperazone/sulbactam	≥64	≥64	–	≥64
Cefepime	≥32	≥32	≥64	≥32
Aztreonam	≥64	≥64	≥64	≥64
Imipenem	≥16	≥16	≥16	≥16
Meropenem	≥16	≥16	–	≥16
Amikacin	≤2(S)	≤2(S)	≤2(S)	≤2(S)
Tobramycin	≥16	≥16	8(I)	8(I)
Ciprofloxacin	≥4	≥4	≥4	≥4
Levofloxacin	≥8	≥8	≥8	≥8
Doxycycline	≥16	≥16	–	≥16
Minocycline	≥16	≥16	–	≥16
Tigecycline	2	2	2(S)	2(S)
Colistin	≤0.5(S)	≤0.5(S)	≤0.5(S)	≤0.5(S)
Trimethoprim/sulfamethoxazole	≤20(S)	≤20(S)	≤20(S)	≤20(S)
CAZ–AVI	S	S	S	S

Broth microdilution and drug sensitivity tests of culture-positive specimens of the 2 cases were performed in accordance with the requirements of the Chinese Clinical and Laboratory Standards Institute. Carbapenemase was detected using the standard paper diffusion method for enzyme inhibitor enhancement testing. The K–B method was used to detect drug sensitivity to CAZ.

CAZ–AVI = ceftazidime–avibactam, MIC = minimum inhibitory concentration.

**Figure 1.**
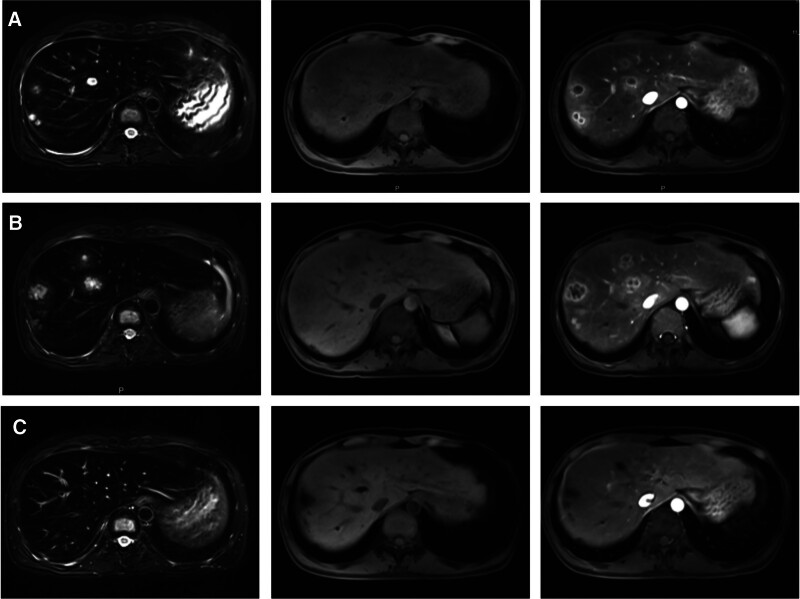
Liver lesions in case 1. (A) Diffuse multiple nodular lesions of varying sizes in the liver with a maximum diameter of approximately 16 mm observed. A hypointense signal on T1WI, halo in the periphery, slightly high signal in the center dots, and hyperintense signal on T2WI are observed. Enhanced scan of the lesion indicated significant obvious enhancement in the periphery of the lesion and no enhancement in the center, clear margins, and abnormal perfusion surrounding the lesion. (B) Progression of the liver lesion is more pronounced than before after 3 weeks of empirical antifungal therapy (maximum diameter of approximately 28 mm with the honeycomb sign present). (C) Significant disappearance of the liver lesions and improvement with CAZ–AVI treatment of CRKP infection (left to right: T2WI, TIWI pre, TIWI post). CAZ–AVI = ceftazidime–avibactam, CRKP = carbapenem-resistant *Klebsiella pneumoniae*.

Day 26 after chemotherapy, the patient again developed fever with a temperature of 38.5 °C. Enhanced MRI of the liver indicated multiple abnormal liver nodules, and hepatic fungal infection was initially considered (Fig. 1). Empirical antifungal therapy (micafungin 150 mg IV q day + posaconazole 10 mL po bid, 3 weeks), was administered, but the lesion remained enlarged after 3 weeks. Ultrasound-guided puncture biopsy of the hepatic lesion was performed, and 2 mL of pus was withdrawn. Histopathology suggested fibrous hyperplasia of vascular tissue and increased infiltration of plasma cells and histiocytes in liver tissue, and an inflammatory lesion was considered. Specific staining did not reveal fungal hyphae or spores. Pathogen culture of the punctured pus indicated CRKP (Table 1). It was clear that the liver lesion was a bacterial liver abscess caused by CRKP. CAZ–AVI 2.5 g IV q8 hours was administered again for 14 days, and body temperature gradually returned to normal without any adverse events.

### 2.2. Case 2

The patient was a 28-year-old male with a history of surgery for traumatic injury of the lower left limb. He was diagnosed with Ph + B-cell ALL in March 2018 and underwent allo-HSCT in April 2019. He experienced recurrence in November 2019 but achieved complete remission through chemotherapy. He underwent maintenance chemotherapy with the VP regimen (Vindesine 4 mg on days 1 and 8 + Dexamethasone 10 mg on days 1–7 and 5 mg on days 8–14) in August 2020. Granulocyte deficiency began on + 11 days after initiation of chemotherapy. The patient developed a fever on + 15 days with *T*_max_ of 38.7 °C. He was administered empirical treatment with meropenem (1 g IV q8 hours, 3 days) and tigecycline (100 mg IV q12 hours, 2 days), but body temperature was not controlled. Blood culture indicated CRKP (drug sensitivity test results shown in Table 1). Antibiotic was switched to CAZ–AVI 2.5 g IV q8 hours, after which the body temperature gradually returned to normal. Granulocyte deficiency lasted for 6 days. CAZ–AVI was discontinued after granulocyte counts returned to normal, and antibiotic regimen was switched to tigecycline (100 mg IV q12 hours, 2 days). The duration for blood culture conversion was 8 days.

**Figure 2.**
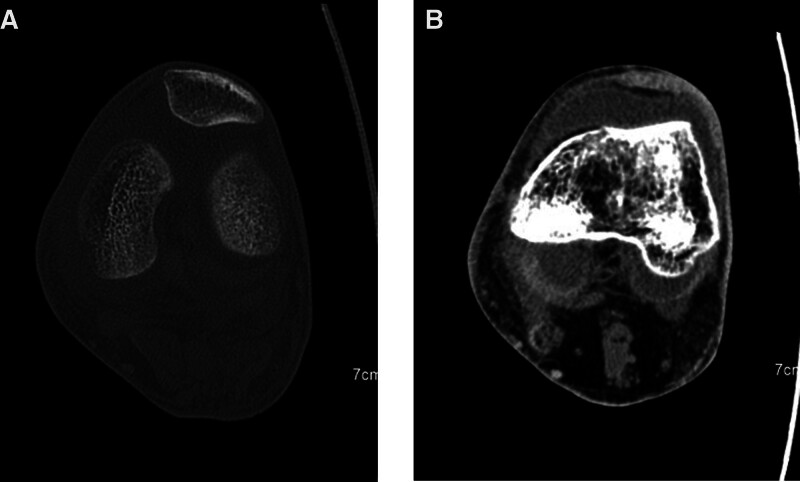
Knee-joint lesions in case 2 of CT scan. (A) Bone destruction seen in the bone window. (B) Fluid effusion and surrounding soft tissue swelling seen in the soft tissue window.

On day 22 after chemotherapy, the patient developed a fever with maximum body temperature of 39 °C again, and CRP increased to 279.3 mg/L. These symptoms were accompanied by left knee redness and swelling, warmth, and pain over an area of approximately 5 × 5 cm, with limited joint mobility. Joint soft tissue infection was considered. CT of the knee indicated joint effusion and swelling of the surrounding soft tissues (Fig. 2). Synovial cavity puncture biopsy was performed, and 20 mL of pus was extracted; the extracted fluid was exudative. Pathogen culture indicated CRKP (drug sensitivity test results shown in Table 1). It was clear that the patient had septic arthritis secondary to CRKP bloodstream infection. CAZ–AVI 2.5 g q8 hours was administered again, and the duration of treatment was 9 days. Symptoms improved after arthrocentesis and antibiotic treatment, with no adverse events.

## 3. Discussion

CRKP poses a serious threat to immunocompromised patients, especially those with HM and who have undergone HSCT.^[4]^ Patients with HM account for 16% to –24% of all patients with CRKP bloodstream infections, with a mortality rate of nearly 60%.^[1,5]^ Risk factors for CRKP infection in patients with HM include previous carbapenem-resistant Enterobacteriaceae colonization or infection, previous use of antibiotics such as carbapenems and compound enzyme inhibitor antibiotics, advanced age, ICU admission, prolonged hospitalization, prolonged (≥7 days) neutrophil deficiency, and allo-HSCT. Early diagnosis and rapid initiation of targeted therapies are critical for immunocompromised hosts with CRKP infections and can improve their prognosis.

Concomitant CRKP bloodstream infection and secondary disseminated organ and tissue infection, such as liver abscess and septic arthritis, is very rare. *K pneumoniae* accounts for 52.4% to 81.7% of the bacteria that cause liver abscesses.^[6]^ CRKP accounts for 3.2% of all *K pneumoniae*,^[7]^ and hypervirulent CRKP has a very high mortality rate, which may pose an urgent future challenge. The pathogens causing septic arthritis are primarily Gram-positive bacteria, especially *Staphylococcus aureus*, and are rarely Gram-negative bacilli with multiple drug-resistant or extensively drug-resistant.^[8]^ Cases of septic arthritis treated with CAZ–AVI were only successful on a case-by-case basis.^[9–11]^ Herein, we described 2 patients with rare disseminated tissue infections. Both cases could have been misdiagnosed as other pathogenic infections, but CRKP was diagnosed through puncture biopsy.

The incidence of misdiagnosis due to deep tissue and organ infections with atypical clinical signs is high.^[12]^ Such patients have a higher probability of severe complications and mortality. Leukopenia causes lack of local pus formation, resulting in atypical clinical symptoms and imaging findings. The detection rate from local pus cultures is higher than that from blood cultures, and next generation sequencing offers the advantage of early detection of pathogens.^[13]^ However, patients with HM often have a risk of bleeding due to disease or treatment factors, which manifest as thrombocytopenia or coagulation abnormalities. Puncture biopsy often exposes such patients to the risk of severe hemorrhage and spread of local infections. We recommend obtaining a tissue specimen as soon as possible when initial treatment is ineffective. This can be combined with microbiological culture and next generation sequencing to rapidly identify the pathogen, which will enable targeted therapy to improve clinical efficacy.

CAZ–AVI is a combination of the third-generation cephalosporin CAZ and the novel β-lactamase inhibitor AVI, which is active against Ambler class A (KPC) and class D (OXA-48) serine carbapenemases but inactive against MBL.^[14]^ It was approved by the China Food and Drug Administration in 2019 for the treatment of complicated urinary tract infections and abdominal infections. In China, the mechanism of CRKP resistance is primarily through KPC-2.^[15]^ Reports on the efficacy of CRKP in disseminated infections are limited, and the pharmacokinetics and pharmacodynamics of CAZ–AVI in liver abscess and septic arthritis and the optimal duration of therapy require further investigation. In patients with HM, the granulocyte deficiency period should be covered at a minimum, but the duration of treatment should be individualized depending on the patient’s clinical response. Due to the limitation of the laboratory at our center, drug resistance gene detection and enzyme analysis were not completed.

CAZ–AVI is well-tolerated clinically, and a systematic evaluation suggested no statistical difference in adverse events between a CAZ–AVI group and control group,^[16]^ with some patients experiencing asymptomatic transaminase elevation and secondary *Clostridioides difficule*-associated diarrhea. However, a safety study of CAZ–AVI by Sternbach et al^[17]^ reached a different conclusion: serious adverse effects associated with CAZ–AVI were significantly higher than those with other antibiotics (primarily carbapenems). No serious adverse effects occurred in the 2 patients in the present study.

In conclusion, disseminated infection secondary to CRKP bloodstream infection in patients with HM is rarely encountered, and puncture biopsy and pathogen identification should be performed as soon as possible for deep organ and joint tissue infections. CAZ–AVI has reliable therapeutic efficacy and few adverse effects in disseminated CRKP infections. However, the number of cases is relatively small, necessitating further validation of this efficacy in large-sample clinical trials.

## Acknowledgments

We are particularly grateful to all the people who have given us help on our article.

## Author contributions

**Conceptualization:** Huifang Jiang.

**Data curation:** Mingxia He, Yuxia Jiang, Haiying Wu.

**Formal analysis:** Mingxia He.

**Funding acquisition:** Haiying Wu.

**Investigation:** Yuxia Jiang, Haiying Wu.

**Methodology:** Xiaofeng Xu.

**Project administration:** Xiaofeng Xu, Huifang Jiang.

**Supervision:** Huifang Jiang.

**Writing – original draft:** Mingxia He.

**Writing – review & editing:** Xiaofeng Xu, Huifang Jiang.

## References

[1] Pouch SM Satlin MJ. Carbapenem-resistant Enterobacteriaceae in special populations: solid organ transplant recipients, stem cell transplant recipients, and patients with hematologic malignancies. Virulence. 2017;8:391–402. 27470662 10.1080/21505594.2016.1213472 PMC5477691

[2] van Duin D Kaye KS Neuner EA Bonomo RA. Carbapenem-resistant Enterobacteriaceae: a review of treatment and outcomes. Diagn Microbiol Infect Dis. 2013;75:115–20. 23290507 10.1016/j.diagmicrobio.2012.11.009 PMC3947910

[3] Zhanel GG Lawson CD Adam H. Ceftazidime–avibactam: a novel cephalosporin/β-lactamase inhibitor combination. Drugs. 2013;73:159–77. 23371303 10.1007/s40265-013-0013-7

[4] van Loon K Voor In ‘t Holt AF Vos MC. A systematic review and meta-analyses of the clinical epidemiology of Carbapenem-resistant Enterobacteriaceae. Antimicrob Agents Chemother. 2018;62:e01730–17. 29038269 10.1128/AAC.01730-17 PMC5740327

[5] Satlin MJ Jenkins SG Walsh TJ. The global challenge of carbapenem-resistant Enterobacteriaceae in transplant recipients and patients with hematologic malignancies. Clin Infect Dis. 2014;58:1274–83. 24463280 10.1093/cid/ciu052 PMC4038783

[6] Siu LK Yeh KM Lin JC Fung CP Chang FY. *Klebsiella pneumoniae* liver abscess: a new invasive syndrome. Lancet Infect Dis. 2012;12:881–7. 23099082 10.1016/S1473-3099(12)70205-0

[7] Yang Q Jia X Zhou M. Emergence of ST11-K47 and ST11-K64 hypervirulent carbapenem-resistant *Klebsiella pneumoniae* in bacterial liver abscesses from China: a molecular, biological, and epidemiological study. Emerg Microbes Infect. 2020;9:320–31. 32037975 10.1080/22221751.2020.1721334 PMC7034084

[8] Urish KL Cassat JE. *Staphylococcus aureus* osteomyelitis: bone, bugs, and surgery. Infect Immun. 2020;88:e00932–19. 32094258 10.1128/IAI.00932-19 PMC7309607

[9] Cani E Moussavi F Ocheretyaner E Sharma R Brown C Eilertson B. Carbapenem-resistant *Klebsiella pneumoniae* vertebral osteomyelitis in a renal transplant recipient treated with ceftazidime–avibactam. Transpl Infect Dis. 2018;20:e12837. 29359842 10.1111/tid.12837

[10] Rico-Nieto A Moreno-Ramos F Fernández-Baillo N. Lumbar arthrodesis infection by multi-resistant *Klebsiella pneumoniae*, successfully treated with implant retention and ceftazidime/avibactam. Rev Esp Cir Ortop Traumatol (Engl Ed). 2018;62:471–3. 29636227 10.1016/j.recot.2018.02.002

[11] De León-Borrás R Álvarez-Cardona J Vidal JA Guiot HM. Ceftazidime/avibactam for refractory bacteremia, vertebral diskitis/osteomyelitis with pre-vertebral abscess and bilateral psoas pyomyositis secondary to *Klebsiella Pneumoniae* carbapenemase-producing bacteria (KPC). P R Health Sci J. 2018;37:128–31. 29905925

[12] Lo JZ Leow JJ Ng PL. Predictors of therapy failure in a series of 741 adult pyogenic liver abscesses. J Hepatobiliary Pancreat Sci. 2015;22:156–65. 25339111 10.1002/jhbp.174

[13] Besser J Carleton HA Gerner-Smidt P Lindsey RL Trees E. Next-generation sequencing technologies and their application to the study and control of bacterial infections. Clin Microbiol Infect. 2018;24:335–41. 29074157 10.1016/j.cmi.2017.10.013 PMC5857210

[14] Ehmann DE Jahić H Ross PL. Avibactam is a covalent, reversible, non-β-lactam β-lactamase inhibitor. Proc Natl Acad Sci USA. 2012;109:11663–8. 22753474 10.1073/pnas.1205073109 PMC3406822

[15] Wei ZQ Du XX Yu YS Shen P Chen YG Li LJ. Plasmid-mediated KPC-2 in a *Klebsiella pneumoniae* isolate from China. Antimicrob Agents Chemother. 2007;51:763–5. 17145797 10.1128/AAC.01053-06 PMC1797727

[16] Zhong H Zhao XY Zhang ZL. Evaluation of the efficacy and safety of ceftazidime/avibactam in the treatment of Gram-negative bacterial infections: a systematic review and meta-analysis. Int J Antimicrob Agents. 2018;52:443–50. 30012440 10.1016/j.ijantimicag.2018.07.004

[17] Sternbach N Leibovici Weissman Y Avni T Yahav D. Efficacy and safety of ceftazidime/avibactam: a systematic review and meta-analysis. J Antimicrob Chemother. 2018;73:2021–9. 29659836 10.1093/jac/dky124

